# The Chinese Catquest-9SF: validation and application in community screenings

**DOI:** 10.1186/s12886-018-0743-0

**Published:** 2018-03-20

**Authors:** Zequan Xu, Song Wu, Wenzhe Li, Yan Dou, Qiang Wu

**Affiliations:** 1 Department of Ophthalmology, Shanghai Jiao Tong University Affiliated Sixth People’s Hospital, No. 600, Yishan Road, Xuhui District, Shanghai, 200233 People’s Republic of China; 2 School of Integrated Traditional and Western Medicine, Anhui University of Traditional Chinese Medicine, No. 103, Meishan Road, Hefei, Anhui 230038 People’s Republic of China; 3 Clinical Medical College, Tianjin Medical University, No. 176 Xueyuan Road, Dagang District, Tianjin, 100270 People’s Republic of China; 4 Department of Foreign Languages, Hainan Medical University, No. 3, College Road, Longhua District, Haikou City, Hainan Province 571100 People’s Republic of China

**Keywords:** Catquest-9SF, Questionnaires, Patient-reported outcomes, Validation, Rasch analysis

## Abstract

**Background:**

The purpose of this study was to validate the Chinese Catquest-9SF questionnaire in community screenings and explore the correlation between Catquest-9SF scores and Lens Opacities Classification System (LOCS) III cataract grading.

**Methods:**

This was a prospective questionnaire validation study. The Catquest-9SF questionnaire was translated into Chinese and was completed by 104 Chinese cataract patients who were diagnosed in community screening. Rasch analysis was used to assess its psychometric properties, and Spearman correlation coefficient was employed to determine the correlation between Catquest-9SF scores and LOCS III cataract grading.

**Results:**

The Catquest-9SF questionnaire demonstrated ordered response categories and unidimensionality (item fit statistics range: 0.70–1.35); the PSI and PR of the category probability curves were 2.00 and 0.80, respectively. There was a fair but statistically significant correlation between Catquest-9SF (Q6, Q7, and Q8) and LOCS III scores and a moderate correlation between Q4 in Catquest-9SF and subcapsular components for the better eye (*r* = − 0.546, *p* < 0.001).

**Conclusion:**

The Chinese version of Catquest-9SF is a valid and reliable questionnaire in community screenings. Thus, this questionnaire may be expected to be an auxiliary tool for preliminary cataract screening use.

## Background

Patient-reported outcomes (PROs), together with Clinician-reported outcomes (CROs) and laboratory tests (or device measurements) are three types of endpoints of diseases. The major representative of formal PROs is reliable and validated multi-item questionnaires [1]. There are many vision-related functional questionnaires [2–4], such as Visual Functioning 14 (VF-14) [5], NEI-visual functioning questionnaire 25 (NEI-VFQ 25) [6] and Catquest nine-item short-form (Catquest-9SF) [7, 8]. Catquest-9SF has been adopted by the International Consortium for Health Outcomes Measurement (ICHOM) to specifically measure the risk factors for and outcomes of cataracts, which are the main global cause of blindness and vision impairment [9].

Originally, Catquest-9SF contained 19 questions, was available in Swedish and was used by the National Swedish Cataract Register to evaluate the visual disability of cataract patients [10]. However, its nine-item short-form Rasch-scaled version (Catquest-9SF) was shown to be more reliable and valid in measuring the visual disability outcomes of cataract surgery [7].

Currently, Catquest-9SF has been translated and culturally adapted, as well as validated, in Australia [11], Germany and Austria [12], Italy [13], and the Netherlands [14], among other countries. Recently, this questionnaire has been assessed by using Rasch analysis in Chinese populations [15, 16].

However, unsolved problems issues arise on the Chinese Catquest-9SF. On the one hand, the results of Chinese Catquest-9SF is still controversial, one study claimed that all nine questions of Chinese Catquest-9SF are valid and reliable [15], while the other study suggested that it is better remove item 7, for item 7 is misfitting [16].

On the other hand, the aim of the study was also to find the direct evidence that the Catquest-9SF would be used as a routine clinical tool in community screening. Previous studies have mainly focused on the validation of cataract surgery candidates in a hospital setting, but a questionnaire valid in a hospital setting serves only as an indirect evidence it is also valid community screening. There is a usually overlooked difference between the two groups: for patients who were diagnosed as cataract in a community screening could have no awareness of their disease while cataract patients who go to a hospital to ask for clinical help usually are fully aware of the troubles which cataract bring to them. And to prove that Catquest-9SF could be used as a routine clinical tool in community screening, the direct evidence on the validity of Catquest-9SF in a community based population are needed. And the function of Catquest-9SF would be extended if it could be used in community screening.

Furthermore, as an indicator of CROs, Lens Opacities Classification System (LOCS) III cataract grading has been widely used to assess lens opacities [5]. Thus, by determining the correlation between Catquest-9SF scores and LOCS III cataract grading, we can further discover whether Catquest-9SF could reflex the opacity of lens.

## Methods

### Catquest-9SF questionnaire

The Catquest-9SF questionnaire was translated from English into Chinese. Five individuals were included on the translation team: one professor of medical English at a medical university; two independent, bilingual native Chinese ophthalmologists; one senior consultant; and one bilingual translation coordinator. The translation procedures were completed via the following steps: 1 The professor of medical English helped to define the conceptual meaning behind each item; 2 The two bilingual native Chinese ophthalmologists translated the Catquest-9SF questionnaire from English into Chinese independently; 3 The senior consultant reconciled the Chinese translations, which were then back translated into English by a third translator; 4 By comparing the original version and the back-translated version, discrepancies between the two versions were identified; 5 The questionnaire was revised and was tested on five other ophthalmologists and five cataract patients to ensure that the items on the questionnaire could be adequately understood; and 6 After thorough discussion of minor revisions, the questionnaire was finalized. In our final version (presented in Table 1), we tried to be more specific in each item to make the questionnaire more understandable to Chinese people, and we also switched the order of Item 7 and Item 8.

**Table 1:** Final version of the Chinese Catquest-9SF and English version of Catquest-9SF

Item	Chinese Catquest-9SF	English Catquest-9SF
Q1	Vision difficulty in everyday life	Vision difficulty in everyday life
Q2	Vision satisfaction in general	Vision satisfaction in general
Q3	Reading text in the newspaper	Reading text in the daily paper
Q4	Recognizing the faces of people around you	Recognizing the faces of people you come across
Q5	Seeing prices of goods when shopping, or descriptions on medicine bottles or bank receipts, electricity bill, water account, etc.	Seeing prices when shopping
Q6	Seeing to walk on uneven ground	Seeing to walk on uneven ground
Q7	Reading text on TV or in movie or on advertising board	Seeing to do handiwork, woodworking, etc.
Q8	Seeing to do delicate work (needlework, handiwork, carpentry, etc.)	Reading text on TV
Q9	Seeing to carry on an activity/hobby you are interested in, such as photography, calligraphy, Mah-jongg playing	Seeing to carry on an activity/hobby you are interested in

The Catquest-9SF questionnaire contains 9 questions. Seven of the questions cover the perceived difficulties in performing daily-life activities, and one of two global questions is about general difficulties in everyday life. The response options are as follows: 1 = very great difficulty; 2 = great difficulty; 3 = some difficulty; 4 = no difficulty; and 5 = cannot decide. There is also one global question about general satisfaction. The response options are as follows: 1 = very dissatisfied; 2 = rather dissatisfied; 3 = fairly satisfied; 4 = very satisfied; and 5 = cannot decide. The response category “cannot decide” is treated as missing data in the analysis [14].

### Data collection

The subject data were collected in the Gumei community (Xuhui District, Shanghai) in community screenings that aimed to identify cataract patients between June 2016 and July 2016. We included patients who were diagnosed as age-related cataracts and willing to participate in this study. All of them received an ophthalmic examination included best-corrected visual acuity, slit-lamp (and LOCS III cataract grading) intraocular pressure and funduscopy. And all of the involved patients completed Catquest-9SF with the help of an ophthalmologist (the coordinator of the translation team). Patients who had difficulty with the Chinese language (Mandarin) and patients who were with diseases that may potentially affect eye sight (have a history of ocular pathology, corneal or intraocular trauma ocular surgery, or with severe subjective dry eye symptoms etc.) and/or a disease that influences daily-life activities (other than cataracts) were excluded. Written informed consent, was obtained from all subjects. And this study was approved by the Office of Research Ethical Committee of Shanghai Jiao Tong University Affiliated Sixth People’s Hospital, The Declaration of Helsinki was strictly followed in all procedures.

### Statistical analysis

#### Rasch analysis

Rasch analysis is a psychometric model widely used in the assessment of questionnaires that measures both person ability and item difficulty on the same scale. In the current study, Rasch analysis was used to determine how well the items (questions) (1), fit the vision function; (2), separated the patients; (3), targeted the patients’ ability. The data from Catquest-9SF were assessed in the Rasch analysis using WINSTEPS software (version 3.72.3, Chicago, IL) with Andrich’s rating scale model. To validate the Chinese version of Catquest-9SF, five key indicators were used, which included the following: (1) Information-weighted (infit) and outlier-sensitive (outfit) mean-square (MnSq) statistics. MnSq values between 0.7 and 1.3 were considered acceptable for unidimensionality [15]. A value > 1.3 implies too much variance, while < 0.7 implies too little variance [14]. (2) Principal component analysis (PCA). PCA of the residuals was performed, which is also an indicator of unidimensionality. Two criteria were used. The first was that the variance explained by the first component should be adequate (> 50%). The second was that the unexplained variance in the first contrast of the residuals should be less than 3.0 eigenvalue units [15]. (3) Category threshold order. The category threshold order, which is reflected by the category probability curves, is an important parameter for demonstrating the usage of response categories, and it is essential for the calculation of person and item calibrations. Disorder thresholds occur when patients have difficulty discriminating between ordered response options. (4) Person separation index (PSI) and person reliability (PR). The PSI and PR are indicators of measurement precision, aiming to reflect the ability to separate people. For a questionnaire with 3 strata, a PSI of 2.0 represents an acceptable level of separation, while 3.00 represents an excellent level, and a PR > 0.8 represents good reliability, while > 0.9 represents excellent reliability (PR range from 0 to 1) [15]. (5) Person-item map. In the present study, a person-item map shows person measures ranked by their ability level and item difficulties ranked by difficulty. It provides a way to visualize how well the items target the ability of the patients.. The person-item map was expressed in logit values,the logit (or log-odds units) is the natural logarithm of the odds of a participant being successful at a specific task or an item being successfully carried out. A more positive value means more visual disability of cataract patients or more difficulties of items. Ideally, the difference between patients and item measure should be approximately 0 logits, which means the visual disability of cataract patients are targeted to difficulties of items. Most research defined that a difference between the mean person and item measure of more than 1.0 logits generally indicates significant mistargeting [13, 15, 16], while less than 1.0 logits generally indicates slight mistargeting. (6) Cronbach’s α. Cronbach’s α is an indicator of reliability that represents how much confidence can be placed in the consistency of the measurement. A Cronbach’s α > 0.8 represents a good consistency, while > 0.9 represents excellent consistency (ranging from 0 to 1).

#### Other statistics

An assessment of Catquest-9SF was also performed by determining the correlation between LOCS III cataract grading and Catquest-9SF scores. Spearman correlation analysis was performed using SPSS (Version 21.0, IBM Corp., Armonk, NY) and was used to determine the relationship. LOCS III is a subjective grading system that has good reproducibility in cataract grading [17]. The correlation was classified as follows [13]: strong, > 0.8, moderate, 0.5–0.8; fair, 0.3–0.5; and poor, < 0.3.

#### Sample size

There is no consistent guidance on the issue of sample size of pre-test of a questionnaire. For an instrument item (question), different studies suggest different participants, including 5-8, [18] 5-15(Health Outcomes Group (2004). Available from: http://www.Healthoutcomesgroup.com/Tables/ translation.html.), 7-10 (Merkus MP, Dekker FW. Kidney Disease Quality of Life-Short-Form: Translation Document. Amsterdam, The Netherlands. 1997.), 8-15 (Evidence Clinical and Pharmaceutical Research (2004). Available from: http://www.evidence-cpr. com/ieo/qlf.html), 10-15, [19] 10-30 (Fayers, P. M., & Machin, D. (2000). Quality of life. Assessment, analysis and interpretation. New York: Wiley.) According to these previous studies, “10 question for an item” was mostly recommended, thus the recommended sample size was 90 (for 9 questions). Besides, the sample size of a previous validation study on Chinese Catquest-9SF was 102 [15]. Considering all mentioned above, we set the sample size as 90-100.

## Results

### Characteristics of the patients

A total of 104 people participated in the study. There were slightly more females (59%), and the median age was 67 (60-87) years. The basic LOCS III scores of the patients are presented in Table 2. The scores of the Chinese Catquest-9SF questionnaire are presented in Table 3.

**Table 2:** LOCS III cataract grading of all 104 participants

LOCS III cataract grading of 104 participants	Better eye (mean ± SD)	Worse eye (mean ± SD)
Cortical	1.87 ± 1.11	2.05 ± 1.21
Posterior subcapsular	1.08 ± 0.58	1.13 ± 0.71
Nuclear colour	2.94 ± 0.84	3.00 ± 0.86
Nuclear opalescence	2.88 ± 0.78	2.99 ± 0.78

**Table 3:** Rasch validation of the Chinese Catquest-9SF

Item	Score (mean ± SD)	Response category “cannot decide” rate	Item difficulty (logit)	Infit MNSQ	Outfit MNSQ	Cronbach’s α after removing the item
Q1	2.89 ± 0.78	0.96%	0.53	0.70	0.74	0.832
Q2	2.77 ± 0.85	1.92%	0.89	0.78	0.79	0.839
Q3	2.86 ± 0.97	0	0.56	1.14	1.09	0.839
Q4	3.36 ± 0.81	0	−0.82	1.25	1.11	0.843
Q5	2.78 ± 0.99	0	0.78	1.07	0.72	0.826
Q6	3.52 ± 0.68	0.96%	−1.27	0.93	0.90	0.834
Q7	3.25 ± 0.82	0	−0.43	0.91	0.82	0.832
Q8	2.84 ± 1.10	6.73%	1.01	0.82	0.68	0.851
Q9	3.80 ± 0.83	18.27%	−1.26	1.10	1.01	0.847

### Unidimensionality

Unidimensionality is an important assumption of Rasch analysis. As mentioned above, MNSQ and PCA are both indicators of unidimensionality.

For the MNSQ, both the “infit” and the “outfit” MNSQ values (presented in Table 3) for each item were acceptable (0.7- 1.3). For PCA, the residuals explained 51.3% (> 50%) of the raw variance, while the unexplained variance in the 1st contrast was 2.0 (< 3.0) eigenvalue units. Both of these outcomes suggested that there was no evidence of multidimensionality.

### Threshold order

**Fig. 1:**
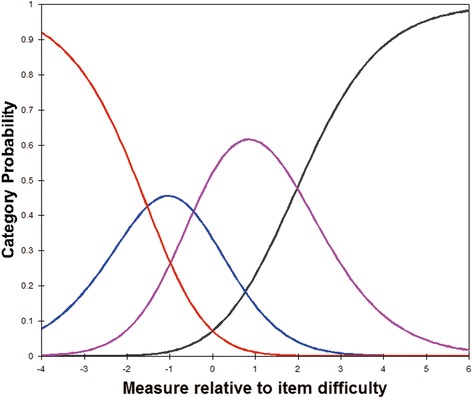
Category probability curves. Category probability curves for the global “difficulties in your daily life” item, which illustrates ordered threshold. The four curves from left to right represent 4 response categories (1 = very great difficulty; 2 = great difficulty; 3 = some difficulty; 4 = no difficulty)

No evidence of disordered thresholds was found in the category probability curves, as the category calibration increased in an orderly way (presented in Fig. 1). Four response categories were found for all items, suggesting three thresholds for each item.

### Separation

Acceptable PSI (2.00) and good PR (0.80) values were respectively found in the analysis, suggesting adequate separation ability for Catquest-9SF.

### Person-item map

**Fig. 2:**
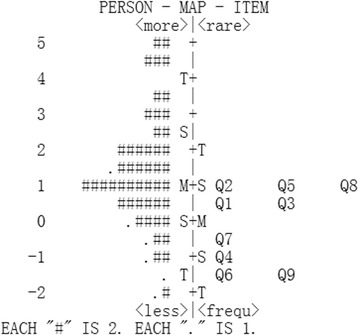
Person-item map

By comparing item difficulty with person ability, the person-item map was used to determine and visualize whether the item difficulties targeted the person abilities in the sample. The person-item map is shown in Fig. 2. On the left dashed line, the less disabled participants (represented by ‘#’ or ‘.’; each ‘#’ and each ‘.’ represents two participants and one participant, respectively) are located at the bottom of the diagram; on the right dashed line, items with lower difficulties are located at the bottom of the diagram. In the present study, item difficulty had a spread from − 1.27 to 1.01 logits (mean value = 0 logits), while patient ability had a spread of − 2.05 to 5.66 logits (mean value = 1.39 logits). Thus, the difference between the item and the person means was 1.39 logits (> 1 logit indicates mistargeting). The mistargeting between patient ability and item difficulty suggested that the specified tasks were relatively easy to perform.

### Reliability: Cronbach’s α

Cronbach’s α was 0.854, and removing any of the items decreased the Cronbach’s α value (presented in Table 3).

### Correlation between LOCS III cataract grading and Catquest-9SF scores

Spearman rank correlation coefficients were used to assess the correlation, and the results are presented in Table 4. A statistically significant (*p* < 0.05 or *p* < 0.01) correlation was found between each question and the LOCS III cataract grading (cortical, posterior subcapsular, nuclear colour and nuclear opalescence). Meanwhile, a moderate correlation was observed between “Recognizing the faces of people around you (Q4)” and posterior subcapsular grading for both the better eye (*r* = 0.532, *p* < 0.0001) and the worse eye (*r* = 0.546, *p* < 0.0001). In addition, fair correlations were observed between Q6, Q7 and Q8 with and cortical grading (*r* > 0.3, *p* < 0.01) for the better eye. Fair correlations were observed between Q1 and nuclear colour and nuclear opalescence grading (r > 0.3, *p* < 0.01) for the better eye.

**Table 4:** Correlation between LOCS III cataract grading and Catquest-9SF

Item	Cortical	Posterior subcapsular	Nuclear colour	Nuclear opalescence
Q1				
Better eye	−0.183	−0.285**	−0.310**	− 0.340**
Worse eye	−0.172	−0.336**	− 0.232*	−0.288**
Q2				
Better eye	−0.085	−0.226*	− 0.084	−0.150
Worse eye	−0.159	−0.287**	− 0.062	−0.101
Q3				
Better eye	−0.179	−0.167	− 0.231*	−0.242*
Worse eye	−0.253**	−0.128	− 0.228*	−0.299**
Q4				
Better eye	−0.202*	−0.532**	− 0.113	−0.029
Worse eye	−0.171	−0.546**	− 0.071	−0.083
Q5				
Better eye	−0.150	−0.472**	− 0.119	−0.097
Worse eye	−0.149	−0.450**	− 0.155	−0.130
Q6				
Better eye	−0.304**	−0.324**	− 0.089	−0.178
Worse eye	−0.233*	−0.297*	− 0.182	−0.259**
Q7				
Better eye	−0.348**	−0.401**	− 0.143	−0.208*
Worse eye	−0.304**	−0.353**	− 0.098	−0.150
Q8				
Better eye	−0.319**	−0.275**	− 0.152	−0.098
Worse eye	−0.269**	−0.173	− 0.118	−0.090
Q9				
Better eye	−0.183	−0.027*	− 0.258**	−0.281**
Worse eye	−0.167	−0.149	− 0.275**	−0.242*
General score				
Better eye	−0.301**	−0.490**	− 0.221*	−0.247*
Worse eye	−0.313**	−0.441**	− 0.203*	−0.236*

**: *p* < 0.01 (two-sided test), significant correlation

*: *p* < 0.05 (two-sided test), significant correlation

## Discussion

The subjects of previous studies on Catquest-9SF validation were mostly hospitalized patients expecting cataract surgery (patients awaiting cataract operation [7, 12–16]) because they were not satisfied with their present vision and had visual disability. The results of our study showed that the Chinese Catquest-9SF was also valid and reliable for assessing cataract patients in community screening and that Catquest-9SF scores have a statistically significant correlation with LOCS III cataract grading. Both of these outcomes suggest that the Chinese Catquest-9SF partly reflects the severity of cataracts in Chinese population-based community screening.

Based on the MNSQ values and PCA, the Chinese Catquest-9SF has demonstrated good unidimensionality. Similar results were found in a study by Lin et al.*....* [15] and for versions in other languages [7, 11–14]. However, in a study by Wang et al, the question about “Seeing to do delicate work (Q8)” was removed from the questionnaire because it was deemed “ambiguous” and failed to demonstrate a good fit (outfit value > 1.3) [16]. Wang et al attributed the misfit to the ambiguity of the word “delicate”. In our study, we elaborated on “delicate work” as “needlework, handiwork, carpentry, etc.” to ensure that patients thoroughly and accurately understood the meaning.

Our study demonstrated an ordered threshold in the category probability curves, which means that patients who responded that they had more visual disability for a certain item indeed had more visual disability for that item than people who claimedthat they had less disability. The combination of a good PSI and good PR suggested that the measurement precision of Catquest-9SF was good, which means that the instrument could accurately distinguish between low and high performers. In the case of Catquest-9SF, this finding specifically means that the measure could accurately distinguish between people with and without cataract-related visual disturbances. Our results were consistent with previous studies, regardless of the Catquest-9SF version used [7, 11–16].

The person-item map showed significant mistargeting (1.39-logit difference in means) of persons and items, suggesting that the specified tasks were relatively easy for the cataract patients, even with decreased visual abilities. Better targeting was found in the study by Wang et al.*..* [16]. This discrepancy may be partially due to the fact that we assessed cataract patients within a community-based population, who tend to have more satisfaction with their present vision and less visual disability in general than hospitalized patients expecting cataract surgery. Meanwhile, mistargeting (1.61 logits) was also found in the study by Lin et al [15] and in studies of a Swedish version (1.95-logit difference in means) [20], Italian version (2.04-logit difference in means) [13], and Dutch version (1.64-logit difference in means) [14]. Thus, the inclusion of additional items that could facilitate better targeting of items for visual abilities should be considered in future studies.

Significant correlation was found between some questions on Catquest-9SF and LOCS III grading, while a moderate correlation was observed for Q4 and posterior subcapsular grading. In addition, fair correlations were observed between “Seeing to walk on uneven ground (Q6)”, “Reading text on TV, in movie or on advertising board (Q7)” and “Seeing to do delicate work (needlework, handiwork, carpentry, etc.) (Q8)” and cortical grading for the better eye. Fair correlations were observed between “Vision difficulty in everyday life (Q1)” and nuclear colour and nuclear opalescence grading for the better eye. In a previous study by Skiadaresi et al.*....,* no correlation was found between the general score on the Italian Catquest-9SF and LOCS III grading [13]. However, in another previous study by Pan et al*,* a significant and moderate correlation was found between the general score on another vision-related functional questionnaire (VF-14) and LOCS III grading (especially nuclear opalescence grading) [5]. Instead of calculating the general score for all questions, we specifically calculated the score for each question. In addition, the sample size of the study by Skiadaresi et al was too small (only 24 patients with nuclear cataracts, 3 with cortical cataracts, and 25 with posterior subcapsular cataracts) to obtain significant results. Thus, Catquest-9SF can reflect lens opacities to some extent.

This study has a few limitations. First, all patients were recruited only from a single community, so larger and more representative samples are needed in future studies. Second, we only investigated the relationship between LOCS III grading and Catquest-9SF, other objective methods, such as lens density and objective scatter index measurement, could be used in future studies. Third, to determine whether Catquest-9SF could be useful as a screening tool, future studies between patients with and without cataract-related visual disturbances might be still needed.

## Conclusion

In conclusion, the Chinese Catquest-9SF is a concise, valid and reliable questionnaire that is easy to understand and quick to complete for Chinese-speaking patients in Chinese community. Moreover, Catquest-9SF scores had a certain correlation with LOCS III grading. Thus, the Chinese Catquest-9SF is expected to be an auxiliary tool for preliminary cataract screening use.
